# Hemolytic Anemia-Related Acute Kidney Injury: A Case Report With Complications Including Posterior Reversible Encephalopathy Syndrome

**DOI:** 10.7759/cureus.44345

**Published:** 2023-08-29

**Authors:** Noman Salih, Numan Ghani, Hidayat Ullah, Izhar Ullah, Abbas Khan, Muhammad Ihtisham

**Affiliations:** 1 General Internal Medicine, Hayatabad Medical Complex Peshawar, Peshawar, PAK; 2 Internal Medicine, Lady Reading Hospital Peshawar, Peshawar, PAK; 3 Internal Medicine, Hayatabad Medical Complex Peshawar, Peshawar, PAK; 4 Internal Medicine, Khyber Girls Medical College, Peshawar, PAK; 5 Internal Medicine, Khyber Medical Center, Peshawar, PAK

**Keywords:** idiopathic hemolytic anaemia, blood transfusions, seizure management, fluids, posterior reversible encephalopathy syndrome

## Abstract

Introduction: The condition known as posterior reversible encephalopathy syndrome (PRES) is characterized by symptoms such as headaches, seizures, and vision problems due to brain swelling, which often can be seen in brain scans. While there have been some cases of PRES linked to conditions such autoimmune diseases and high blood pressure, we're sharing a unique case here. Our case involves severe kidney damage caused by idiopathic hemolytic anaemia. The patient also experienced loss of consciousness, seizures, and headache. Brain scans confirmed the signs of PRES. We managed to help the patient recover fully through careful treatment, including fluids, managing seizures, and transfusions.

Case details: Our patient was dealing with severe kidney damage from idiopathic hemolytic anaemia. They had episodes of loss of consciousness, seizures, and headaches. Brain scans showed that they had PRES.

Diagnosis and treatment: We found out that the patient had severe kidney damage because of hemolytic anaemia, and she also had PRES. We treated her by giving fluids, managing her seizures, and doing blood transfusions, along with other supportive care.

Conclusions: With our treatment, the patient got better, her neurological symptoms improved, and her brain scans showed fewer signs of PRES. This case tells us something interesting - sometimes, anaemia can lead to rare neurological problems like PRES. We need to be aware of these possibilities to help patients better. Our successful treatment in this case emphasizes how important quick and comprehensive care can be for good outcomes.

## Introduction

This case sheds light on a special condition called posterior reversible encephalopathy syndrome (PRES), which brings together symptoms like seizures, confusion, odd vision changes, and headaches. An interesting feature of PRES is that it causes swelling in a specific part of the brain called the parieto-occipital white matter. It's important to note that not a lot of research has been done on PRES in children, and most of the information we have comes from individual case reports or small groups of cases [1]. PRES is not just about its symptoms; it's also linked with a type of kidney problem called acute kidney injury (AKI). This kidney issue can happen when red blood cells break down in the bloodstream, leading to problems like inflammation and blockages in the kidney tubes [2]. Anaemia, caused by low levels of haemoglobin in the blood, makes people more likely to have kidney problems [3]. This is especially true when people with anaemia also have hidden kidney issues. Another thing that's interesting is the connection between AKI caused by red blood cell breakdown and PRES. When both these problems occur together, it makes the situation more complicated and harder to understand. People with chronic kidney disease (CKD), a long-term kidney problem, have a higher chance of getting PRES because they often face things like high blood pressure and kidney waste buildup [4]. PRES shows up as a mix of symptoms like headaches, seizures, vision problems, and trouble thinking. Despite all this, we still don't fully understand why PRES happens. This case helps us see how PRES and kidney problems are connected, adding to the puzzle of how they work together.

## Case presentation

We present the case of a 16-year-old female who was admitted to the emergency department in an unconscious state. The patient's caregivers reported a three-day history of syncope, accompanied by a low-grade fever and generalized seizures. Of clinical significance, she had a documented medical history of idiopathic anaemia, necessitating multiple blood transfusions. Additionally, she had previously encountered acute kidney injury episodes attributed to hemolysis-induced nephrotoxicity. An extensive review of the family history did not reveal any known genetic disorders or recurrent medical conditions related to anaemia or renal dysfunction. The patient’s parents reportedly enjoyed unremarkable health statuses.

Upon initial evaluation, the patient exhibited marked pallor and unresponsiveness. Subsequently, she experienced two seizures, prompting prompt intervention with anticonvulsant medications, leading to termination of the seizures and gradual improvement in her Glasgow Coma Scale (GCS). Her baseline vital signs remained within acceptable ranges, albeit with an episode of transient hypertension (150/90 mmHg). Laboratory investigations given in Table 1 demonstrated leukocytosis, elevated urea and creatinine levels, and other unremarkable blood parameters except for the normal findings of creatine kinase (CK) and CK isoenzyme assays, which were performed to exclude myoglobinuric kidney injury. Her autoimmune profile was done along with Coombs and indirect Coombs tests which were normal. A urine routine investigation (R/E) was done which didn't reveal any infection. Notably, the patient displayed no evidence of physical trauma or strenuous exertion to warrant myoglobinuria. Also, thrombotic thrombocytopenic purpura (TTP) was ruled out based on her normal physical findings and normal platelets. Her arterial blood gases showed mild metabolic acidosis with respiratory compensation (ABGs).

**Table 1:** Baseline investigations Abbreviations: WBC, white blood cell; Hb, haemoglobin; PLT, platelets; Na, sodium; K, potassium; Cl, chloride; Creat, creatinine; CK, creatinine kinase; CK, creatinine isoenzyme; BNP, brain natriuretic peptide; AST, aspartate aminotransferase; ALT, alanine aminotransferase; LDH, lactate dehydrogenase

Test	Absolute value	Reference value value
WBC (/µl)	13000	4000-11000
Hb (gr/dl)	7.1	12.5-16.5
PLT (/µl)	367000	150000-400000
Na (mmol/l)	136.5	135-145
K (mmol/l)	3.7	3.5-5.1
Cl (mmol/l)	98	96 - 112
Bilirubin (mg/dl)	1	0.1-1
AST (U/L)	31	8-33
ALT (U/L)	28	7-35
Creat (mg/dl)	2.2	0.7 to 1.3
CK (U/L)	58	30 to 145
BUN (mg/dL)	60	7-20
CK isoenzyme (IU/L)	21	5-25
Myoglobin (ng/mL)	56	25-72
BNP (pg/mL)	48	Less than 100
Lactate (mmol/L)	0.4	less than 1.0
LDH (IU/L)	840	143-370

**Figure 1:**
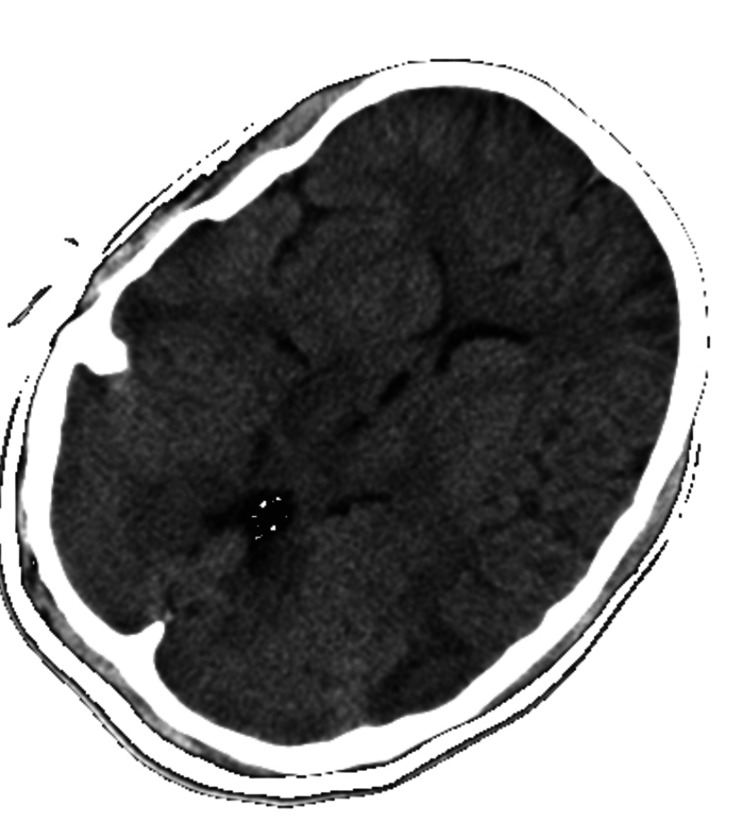
Normal CT brain

Neuroimaging, including a cranial CT scan, revealed no gross intracranial pathology as shown in Figure 1. Subsequent cerebrospinal fluid (CSF) analysis through lumbar puncture exhibited normal parameters, effectively ruling out central nervous system (CNS) inflammation. Electrophysiological assessment in the form of an electroencephalogram (EEG) yielded unremarkable findings.

**Figure 2:**
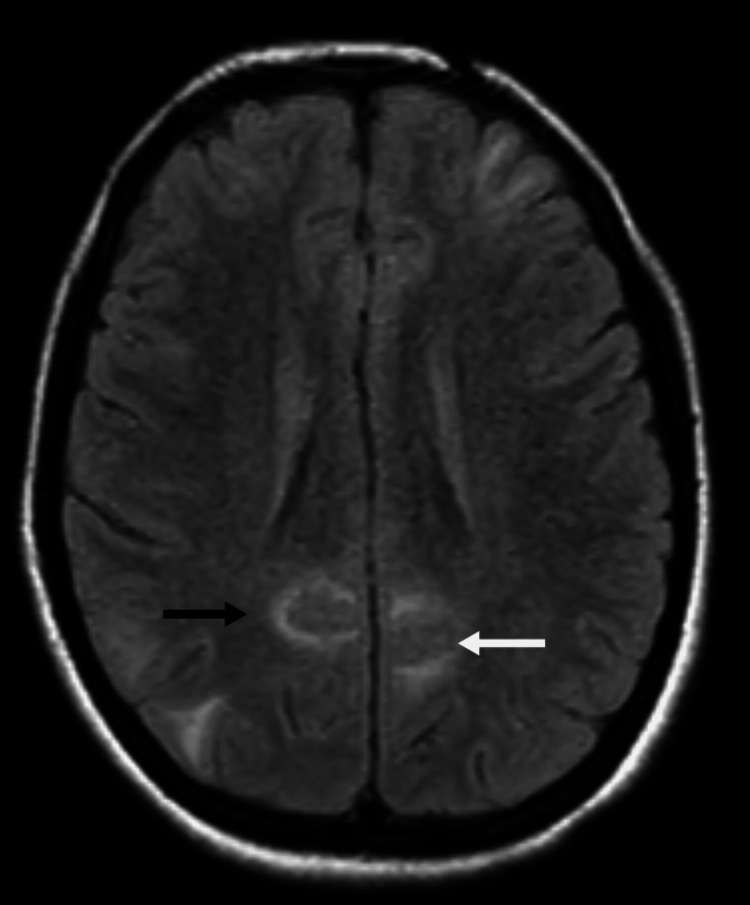
Prominent signals observed in the white matter of the cortical and subcortical regions in both the frontoparietal and occipital region shown by black and white arrows

**Figure 3:**
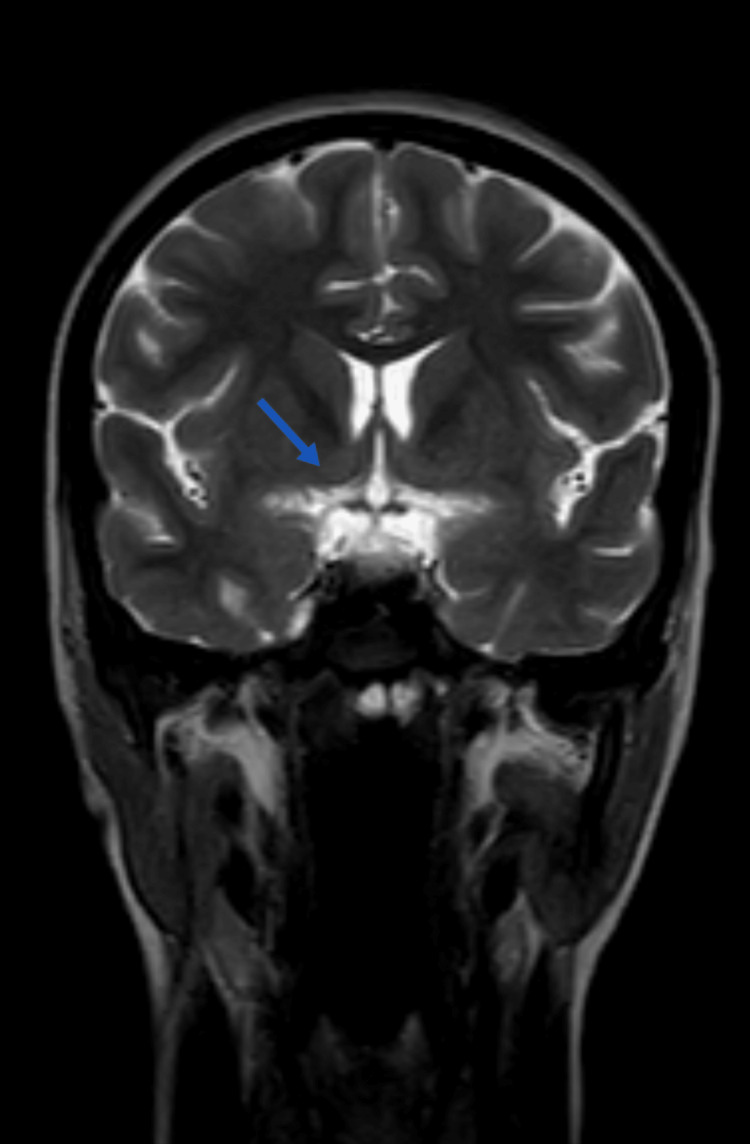
Bright sights shown by blue arrow

To further investigate a potential viral encephalitic aetiology, a cerebral MRI was conducted in conjunction with CSF analysis. CSF was normal while MRI showed the following findings; prominent signals were observed in the white matter of the cortical and subcortical regions in both the frontoparietal, occipital, and temporal lobes on both sides of the brain. These signals appear brighter than usual on T2 and FLAIR images, and there was no indication of restricted diffusion. The ventricular system seemed to be in a normal state. The interhemispheric fissure was aligned with the midline, and the typical sulcation of the cerebral cortex was present without any anomalies. No irregularities were detected in the basal ganglia, internal capsule, corpus callosum, or thalamus. The sella and pituitary regions exhibited no abnormalities, and structures surrounding the sella were within the expected range. The cerebellum displayed a normal appearance. Both the 7th and 8th nerve complexes were normal on both sides. The area around the cerebellopontine angle appears regular on each side. The internal acoustic meatus showed no signs of deviation from the norm. The mastoid air cells exhibited the anticipated characteristics. Additionally, the cavernous, sagittal, straight, and transverse sinuses were all within normal parameters. In conclusion, the bright signals observed in the white matter of the cortical and subcortical regions on T2 and FLAIR images in the bilateral frontoparietal, occipital, and temporal lobes suggested the presence of posterior reversible encephalopathy syndrome (PRES). Images are shown in Figures 2-3.

In terms of therapeutic intervention, a comprehensive strategy was implemented to address both renal and neurological concerns. The management plan encompassed rigorous hydration, diuresis, pH normalization, and general supportive care. Gradual amelioration of symptoms ensued, culminating in clinical stability after a span of three days. The recovery phase was accompanied by the emergence of headaches and visual disturbances, for which specific interventions were tailored. Serial monitoring of renal function parameters, including creatinine and blood urea nitrogen (BUN) levels, evidenced gradual normalization in tandem with daily urine volume increments. Concurrently, antihypertensive measures involving calcium channel blockade (amlodipine) facilitated effective blood pressure control. A 10-day inpatient treatment course yielded favourable outcomes, as evidenced by improved vital signs at the one-week follow-up visit. At the three-month assessment mark, the patient exhibited a complete resolution of symptoms, characterized by the absence of residual renal or neurological deficits.

In summation, this case underscores the intricacies of diagnostic dilemmas compounded by enigmatic clinical presentations. The seamless collaboration of various medical disciplines facilitated the accurate diagnosis and tailored management of this challenging scenario. The patient's trajectory exemplifies the fortitude of both medical science and human resilience in surmounting formidable clinical challenges.

## Discussion

The clinical and radiological condition known as posterior reversible encephalopathy syndrome (PRES) presents a unique set of challenges in its diagnosis and management. PRES is characterized by a constellation of symptoms, including headache, erratic mental status, seizures, visual abnormalities, and distinct radiological findings within the posterior cerebral white matter regions [5]. It is worth noting that while the majority of cases exhibit bilateral changes, approximately 5% of cases manifest unilateral alterations [6]. Despite its name, PRES does not always exclusively involve the posterior white matter, and its reversibility is not guaranteed [7].

Diagnostic methods for PRES can be confounding. Electroencephalography and cerebrospinal fluid analysis often prove ineffective in establishing a definitive diagnosis. Interestingly, a singular case report even highlighted lumbar puncture as a potential trigger for PRES [8]. The complex underlying pathophysiology is still being elucidated, although vascular endothelial dysfunction emerges as a common factor in various conditions associated with PRES. This dysfunction disrupts cerebral autoregulation, leading to the emergence of vasogenic oedema.

A comprehensive understanding of the relevant medical literature provides critical insights into the nature of PRES. Bartynski's series offers a comprehensive overview, shedding light on the diverse clinical presentations and radiological features that can deviate from the conventional posterior white matter involvement [9,10]. Despite the heterogeneous underlying conditions, the thread linking many of them is vascular endothelial dysfunction. This dysfunction's role in impairing cerebral autoregulation and causing vasogenic oedema underscores its significance.

The distinctive predilection of PRES for parieto-occipital regions within the brain is hypothesized to arise from the lower levels of sympathetic innervation in the posterior cerebral circulation [11]. Furthermore, the relatively loose structure of white matter compared to the cortex could render these regions more vulnerable to hydrostatic oedema [12].

In the specific case at hand, the pathogenesis of PRES becomes apparent. Acute hypertension led to an increase in cerebral blood flow that surpassed the cerebrovascular system's regulatory capabilities, ultimately resulting in brain tissue oedema. This was further exacerbated by the endothelial cerebrovascular dysfunction triggered by the circulation of both endogenous and exogenous toxins. The patient's consciousness remained intact, serum lactate levels were within the normal range, and there were no signs of systemic illness. Crucially, cerebrospinal fluid analysis and EEG tests returned with normal results. Viral encephalitis was effectively ruled out based on these findings. Differential diagnoses such as acute toxic encephalopathy, posterior circulation embolism, venous sinus thrombosis, and demyelinating illnesses were also meticulously considered [13].

## Conclusions

In conclusion, the complexities of diagnosing and managing posterior reversible encephalopathy syndrome are evident in this case. It underscores the need to recognize diverse clinical presentations and radiological features, challenging the assumption of exclusive posterior white matter involvement. The pivotal role of vascular endothelial dysfunction in disrupting cerebral autoregulation and inducing vasogenic edema is a central theme. Clinicians should maintain a high index of suspicion for PRES, particularly in cases of acute hypertension and cerebral edema where alternative diagnoses have been excluded. This case serves as a reminder of the intricate nature of neurological disorders, highlighting the importance of comprehensive evaluation and differential diagnosis.
